# Probiotic *Lactobacillus* Species and Their Biosurfactants Eliminate Acinetobacter baumannii Biofilm in Various Manners

**DOI:** 10.1128/spectrum.04614-22

**Published:** 2023-03-15

**Authors:** Mona Mohamed Al-Shamiri, Jingdan Wang, Sirui Zhang, Pu Li, Woodvine Otieno Odhiambo, Yanjiong Chen, Bei Han, E. Yang, Meng Xun, Lei Han, Shaoshan Han

**Affiliations:** a Department of Microbiology and Immunology, School of Basic Medical Sciences, Xi’an Jiaotong University Health Science Center, Xi’an, China; b School of Public Health, Xi’an Jiaotong University Health Science Center, Xi’an, China; c Department of Hepatobiliary Surgery, First Affiliated Hospital of Xi’an Jiaotong University, Xi’an, China; Weizmann Institute of Science

**Keywords:** *Acinetobacter baumannii*, biofilm, probiotic *Lactobacillus*, lactic acid, acetic acid

## Abstract

Acinetobacter baumannii is a critical biofilm-forming pathogen that has presented great challenges in the clinic due to multidrug resistance. Thus, new methods of intervention are needed to control biofilm-associated infections. In this study, among three tested *Lactobacillus* species, Lactobacillus rhamnosus showed significant antimaturation and antiadherence effects against A. baumannii biofilm. Lactic acid (LA) and acetic acid (AA) were the most effective antibiofilm biosurfactants (BSs) produced by L. rhamnosus. This antibiofilm phenomenon produced by LA and AA was due to the strong bactericidal effect, which worked from very early time points, as determined by colony enumeration and confocal laser scanning microscope. The cell destruction of A. baumannii appeared in both the cell envelope and cytoplasm. A discontinuous cell envelope, the leakage of cell contents, and the increased extracellular activity of ATPase demonstrated the disruption of the cell membrane by LA and AA. These effects also demonstrated the occurrence of protein lysis. In addition, bacterial DNA interacted with and was damaged by LA and AA, resulting in significantly reduced expression of biofilm and DNA repair genes. The results highlight the possibility and importance of using probiotics in clinical prevention. Probiotics can be utilized as novel biocides to block and decrease biofilm formation and microbial contamination in medical equipment and during the treatment of infections.

**IMPORTANCE** A. baumannii biofilm is a significant virulence factor that causes the biofilm colonization of invasive illnesses. Rising bacterial resistance to synthetic antimicrobials has prompted researchers to look at natural alternatives, such as probiotics and their derivatives. In this study, L. rhamnosus and its BSs (LA and AA) demonstrated remarkable antibiofilm and antimicrobial characteristics, with a significant inhibitory effect on A. baumannii. These effects were achieved by several mechanisms, including the disruption of the cell envelope membrane, protein lysis, reduced expression of biofilm-related genes, and destruction of bacterial DNA. The results provide support for the possibility of using probiotics and their derivatives in the clinical prevention and therapy of A. baumannii infections.

## INTRODUCTION

Acinetobacter baumannii is an important opportunistic pathogen (1). It has been characterized as one of the top seven pathogens threatening the health care delivery system (2, 3). This pathogen has various virulence characteristics contributing to its pathogenicity, including the ability to form biofilms, which is critical for the organism's survival in hostile environments (4, 5) and thus increases the risk of bacterial infections. A. baumannii can resist several antibiotic classes and can thrive in a range of hospital conditions (6). As a result of the strong biofilm formation and acquired drug resistance of A. baumannii, there is an urgent need to develop new treatments to prevent and control biofilm-associated infections caused by multidrug-resistant (MDR) A. baumannii.

Probiotics have recently gained popularity as a safe and helpful treatment (7, 8). Probiotics are a diverse group of lactic acid bacteria (LAB) genera, and *Lactobacillus* is the most beneficial genus (9, 10). Recently, attention has been directed toward the production of biosurfactants (BSs) using various probiotic bacteria, including LAB, with which the growth of pathogenic microbes may be antagonized (11). BSs isolated from LAB comprise various combinations of proteins, polysaccharides, and organic acids (12). In addition, BSs have multiple benefits, including reduced toxicity, high stability, and activity in a wide range of pHs and temperatures (13). Moreover, BSs may have antimicrobial and antiadhesive properties and thus could be used to prevent biofilm formation (14, 15). Since the current usage of probiotics and BSs is limited, this study aimed to determine whether Lactobacillus rhamnosus, Lactobacillus reuteri, and Lactobacillus acidophilus, and their associated BSs, can inhibit the development of biofilms by A. baumannii. Moreover, we sought to elucidate the mechanism of BSs as potential alternatives to antibiotics for preventing biofilm formation.

## RESULTS

### Antimaturation and antiadherent effects of probiotics.

**FIG 1:**
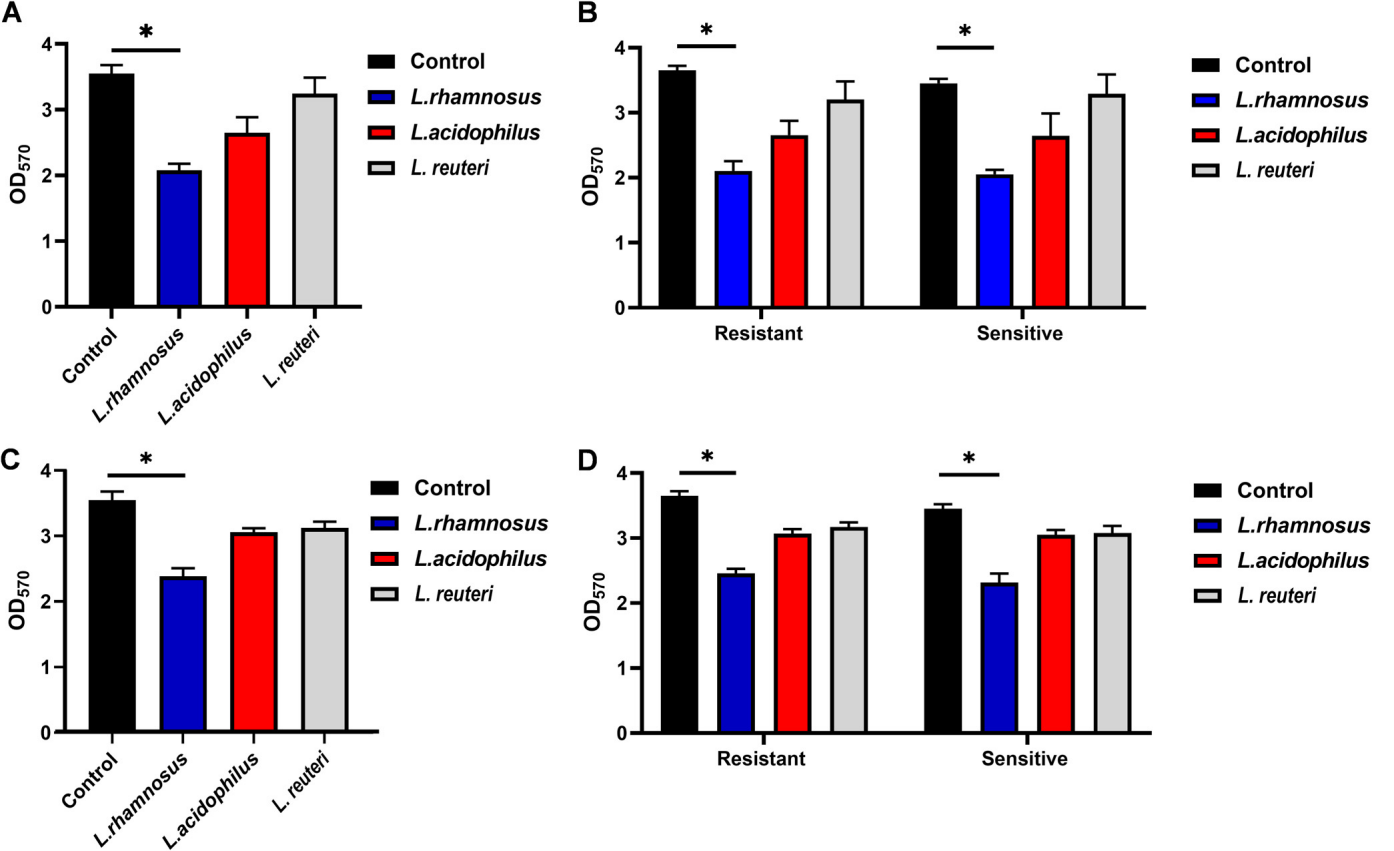
Antimaturation and antiadherent effects of L. rhamnosus, L. acidophilus, and L. reuteri against the biofilm formation of A. baumannii isolates in planktonic culture. (A) Antimaturation effects of all isolates; (B) antimaturation effects of resistant and sensitive isolates; (C) antiadherent effects of all isolates; and (D) antiadherent effects of resistant and sensitive isolates. The results are expressed as means ± standard deviations (SD). *, *P < *0.05.

The antimaturation and antiadherent abilities of L. rhamnosus, L. reuteri, and L. acidophilus were examined by the absorbance of crystal violet by the A. baumannii biofilm. As shown in Fig. 1A, probiotics have the potential to disrupt mature biofilms, as well as to inhibit the biofilm development of A. baumannii. After coculture with L. rhamnosus, L. reuteri, and L. acidophilus, the optical density at 570 nm (OD_570_) of the A. baumannii biofilms decreased from 3.55 to 2.07, 2.64, and 3.24, respectively. Similar results were observed when resistant and sensitive isolates were detected separately (Fig. 1B). L. rhamnosus showed the strongest antimaturation effect against the biofilm of A. baumannii (*P < *0.05).

At the same time, the antiadherent effects of the three *Lactobacillus* strains were noted. The OD_570_ of A. baumannii biofilms decreased to 2.38, 3.05, and 3.12 after treatment with L. rhamnosus, L. acidophilus, and L. reuteri, respectively (Fig. 1C). Comparable outcomes were demonstrated when resistant and sensitive isolates were detected separately, as illustrated in Fig. 1D. Among the three *Lactobacillus* spp., L. rhamnosus had the strongest antiadherent effect (*P < *0.05).

### Antibiofilm effects of BSs derived from L. rhamnosus.

**FIG 2:**
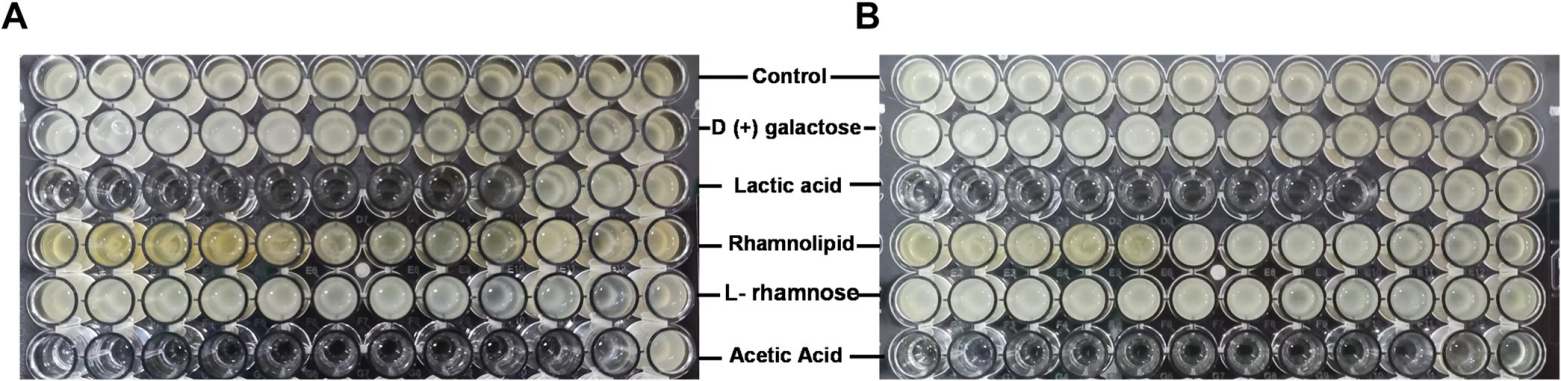
Antimaturation (A) and antiadherent (B) effects of BSs derived from L. rhamnosus on A. baumannii biofilm. The broth microdilution method was used to detect the effects of different BSs. d-(+)-Galactose, lactic acid (LA), rhamnolipid, and l-rhamnose monohydrate were tested at concentrations of 500, 250, 125, 62.5, 31.2, 15.6, 7.8, 3.9, 1.95, 0.97, 0.48, and 0.24 mg/mL, and acetic acid (AA) was tested at concentrations of 100, 50, 25, 12.5, 6.25, 3.125, 1.56, 0.78, 0.39, 0.195, 0.097, and 0.048%.

Since L. rhamnosus exhibited a strong inhibition effect on the maturation and adherence of A. baumannii biofilm, its BSs may exert similar functions. Therefore, the most commonly reported BSs derived from L. rhamnosus were tested for their ability to clear bacterial biofilm, including d-(+)-galactose, l-rhamnose monohydrate, lactic acid (LA), and rhamnolipids in the range of 500 to 0.24 mg/mL and acetic acid (AA) in the range of 100 to 0.097%. Among these BSs, LA and AA exhibited high antimaturation (Fig. 2A) and antiadherent (Fig. 2B) effects against A. baumannii biofilm. LA completely disrupted an already-formed biofilm at 3.9 mg/mL and inhibited the formation of biofilm at an even lower concentration of 1.95 mg/mL. AA displayed both functions at the low concentration of 0.39%. However, the other three BSs failed to destroy A. baumannii biofilm at even the highest concentrations. Thus, LA and AA were tested in subsequent experiments.

### Antibiofilm and antibacterial effects of LA and AA.

**FIG 3:**
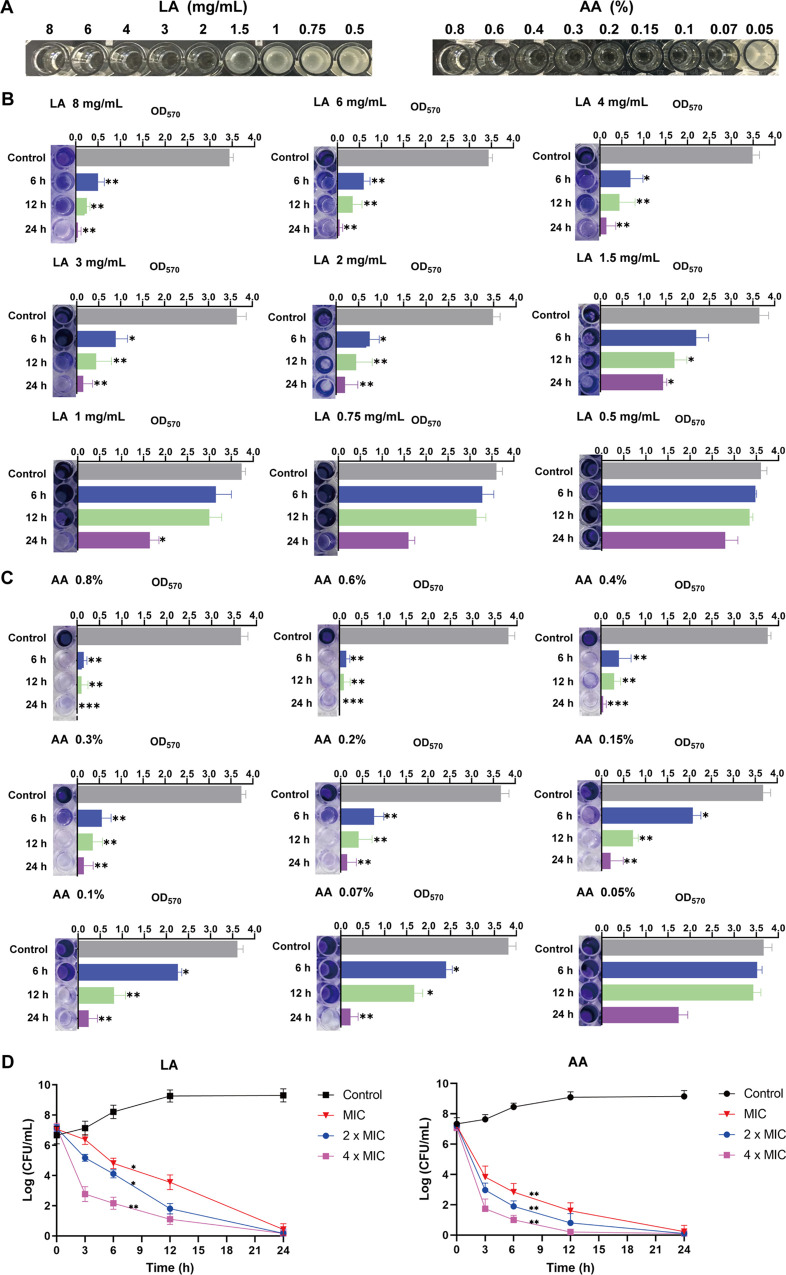
Antibiofilm and antibacterial effects of LA and AA. (A) MIC determinations for LA and AA using the broth microdilution method. (B and C) Antibiofilm effects of LA and AA were observed at different time points. (D) Survival curve of A. baumannii after treatment with LA and AA. All of the experiments were repeated in triplicate, and the results are expressed as means ± SD. *, *P < *0.05; **, *P < *0.01; ***, *P < *0.001.

In order to find an appropriate concentration to inhibit biofilm, the MIC values of LA and AA were first tested. As shown in Fig. 3A, LA and AA were effective in preventing the growth of planktonic A. baumannii isolates at concentrations of 2 mg/mL and 0.07%, respectively.

Subsequently, we intended to check the antibiofilm ability of LA and AA over a shorter duration; thus, the experiment was performed at the time points of 6 h, 12 h, and 24 h. As expected, the longer the biofilm was incubated with LA and AA, the stronger the observed antibiofilm effects. Nevertheless, above the MIC values of LA and AA, a significant reduction in biofilm was shown for both 6 h and 12 h (Fig. 3B and C).

The death of bacterial cells is closely related to the clearance of biofilm. Thus, the bactericidal efficiency of LA and AA was determined by evaluating the number of live cells after treatment for different durations. The numbers of A. baumannii were reduced by both LA and AA with different MICs from early time points. The higher the concentration used, the fewer live cells remained. The number of bacteria decreased remarkably in a time-dependent manner. In particular, after 24 h of treatment, almost no live bacteria could be detected at any concentrations for both LA and AA.

**FIG 4:**
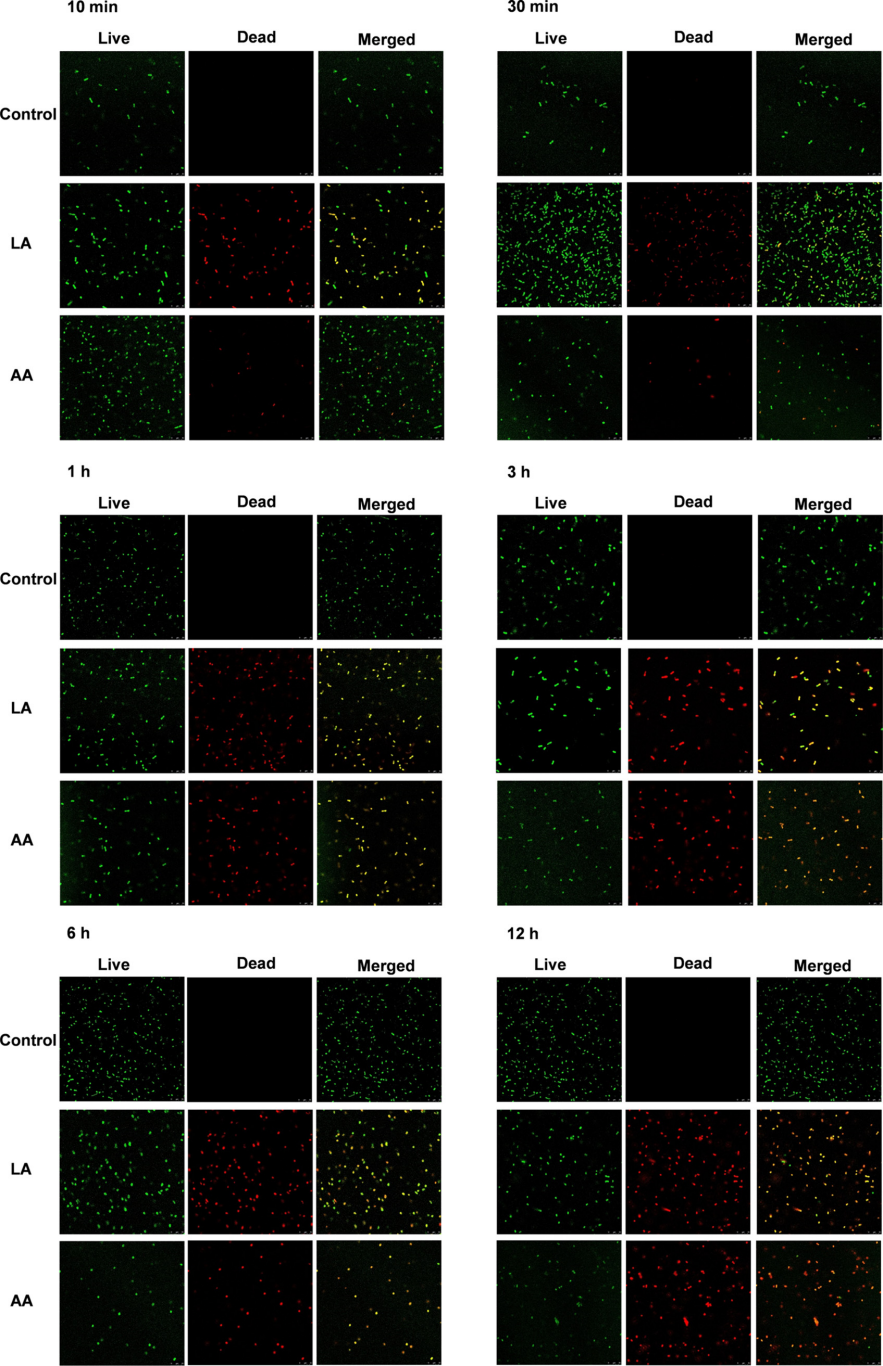
CLSM images of controls and A. baumannii treated with LA and AA at different time points of 10 min, 30 min, 1 h, 3 h, 6 h, and 12 h. Images were captured at 63× magnification.

The confocal laser scanning microscope (CLSM) results further confirm that LA and AA had strong bactericidal effects (Fig. 4). A. baumannii cells remained alive for all of the time points of detection in the control group. Surprisingly, a large number of damaged cells were captured after very short treatment with both LA and AA for only 10 min. Additionally, dead cells were noticed from 30 min. Furthermore, over 86% and 94% of bacterial cells were broken after treatment for 1 to 6 h with LA and AA, respectively. In addition, at 12 h posttreatment, the A. baumannii cells were stained almost completely red and exhibited extremely weak green fluorescence.

### Inhibition of twitching motility by LA and AA.

**FIG 5:**
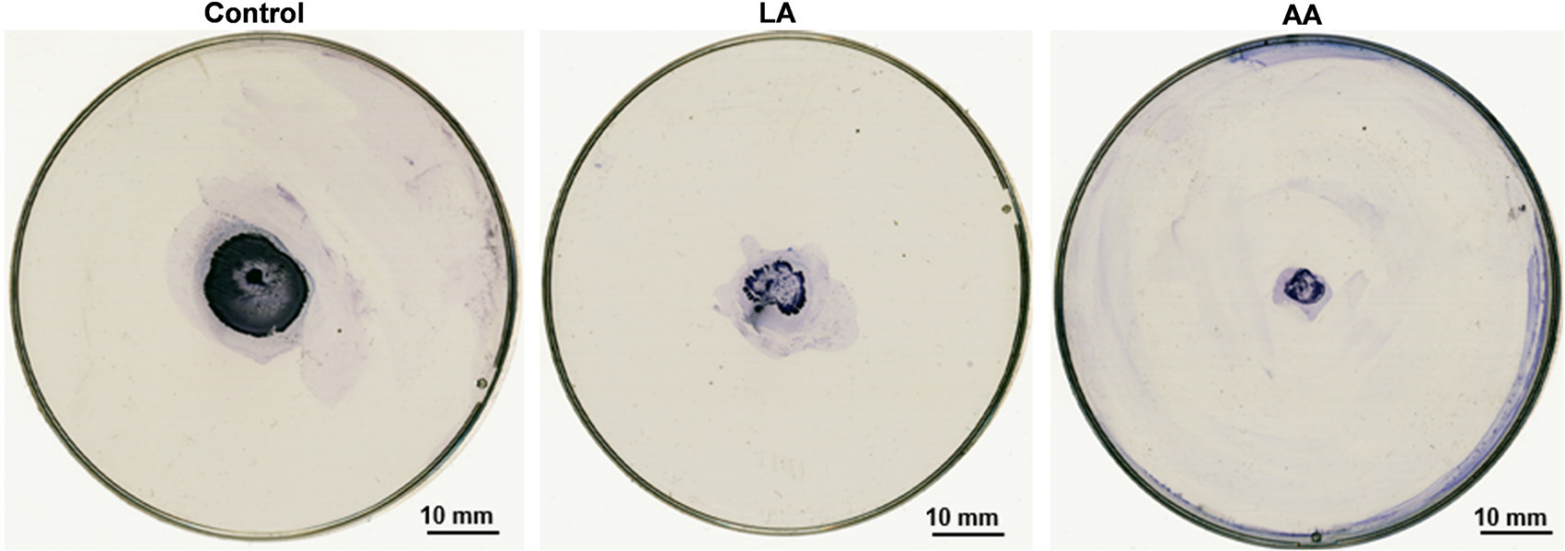
Inhibition of twitching motility in A. baumannii at 1/2 the MICs of LA and AA in MH medium. The twitching motility was reduced in the presence of LA and AA compared with the control.

Since twitching motility is a kind of reflection of bacterial activity, the influence of LA and AA on this ability was also determined using the concentration of 1/2 MIC. Twitching motility was assayed based on the ability of the cells to spread on polystyrene petri dishes. As detailed in the following section, the diameter of the twitching motility zone was significantly reduced from 20 mm to 11 mm (*P < *0.01) after treatment with LA. In addition, AA treatment resulted in a greater reduction in the zone to 6 mm (*P < *0.001) (Fig. 5).

### Effects of LA and AA on cell morphology.

**FIG 6:**
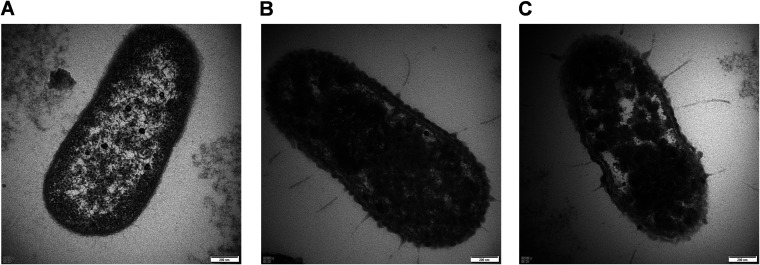
TEM images of A. baumannii. (A) A cell of untreated A. baumannii was intact, with a well-defined cell membrane and homogenous cytoplasm. (B and C) After treatment with LA and AA at the respective MICs for 3 h, damage was observed in both the cell envelope and cytoplasm.

Transmission electron microscopy (TEM) was further performed to investigate the structural changes in A. baumannii affected by LA and AA. As shown in Fig. 6A, normal A. baumannii showed an intact and tough cell envelope, which was evenly distributed around the cytoplasm. However, bacterial cells were significantly damaged after exposure to LA and AA for 3 h. High-density condensed substances were observed in the cytoplasm, with some parts harboring coagulated materials after treatment with LA. Simultaneously, the cell envelope was disrupted and disintegrated. As a result, the cell surface was covered by appendages that formed a filament-like structure, which were sites of the excessive leakage of essential cytoplasmic contents (Fig. 6B). A similar phenomenon was observed for AA. However, stronger clotting of cytoplasmic components was noticed for AA compared with LA in the same duration of time, which led to higher heterogeneous cytoplasmic density (Fig. 6C).

### Effects of LA and AA on cell membrane permeability.

**FIG 7:**
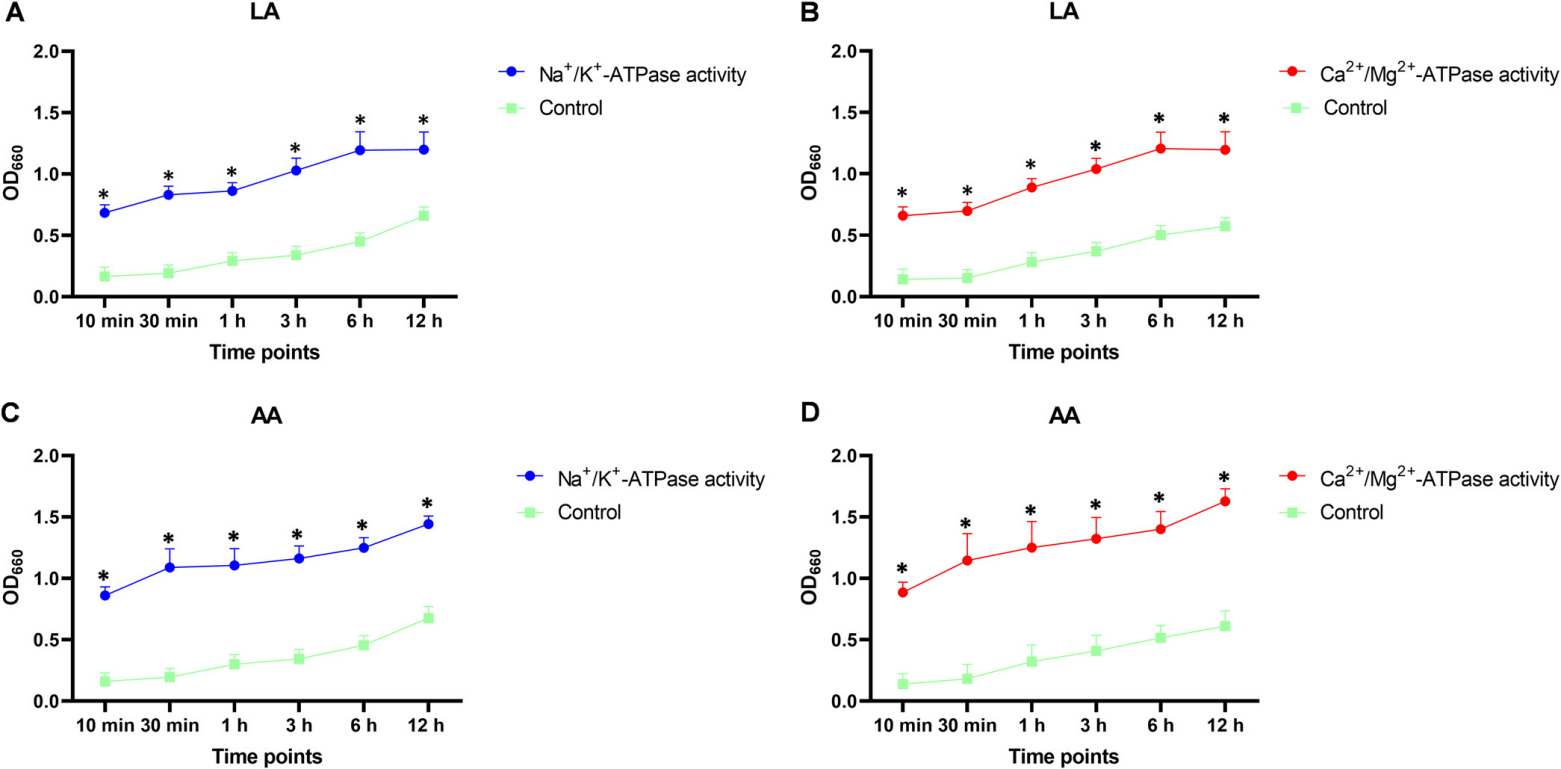
Effects of LA and AA on ion pump activity. (A and B) Effect of LA on the activity of Na^+^/K^+^-ATPase (A) and Ca^2+^-Mg^2+^-ATPase (B). (C and D) Effect of AA on the activity of Na^+^/K^+^-ATPase (C) and Ca^2+^-Mg^+^-ATPase (D). All of the experiments were repeated in triplicate, and the results are expressed as means ± SD. *, *P < *0.05.

Ion pumps, such as Na^+^/K^+^-ATPase and Ca^2+^/Mg^2+^-ATPase, are important proteins located in the plasma membranes of cells (16, 17). Apparent increases in the activity of Na^+^/K^+^-ATPase and Ca^2+^/Mg^2+^-ATPase were detected after exposing A. baumannii strains to both LA and AA at the respective MICs. Treatment with LA or AA caused a significant increase (*P < *0.05) in the activity of both ATPases with different durations of treatment in comparison with the control (Fig. 7).

### Effects of LA and AA on A. baumannii protein.

**FIG 8:**
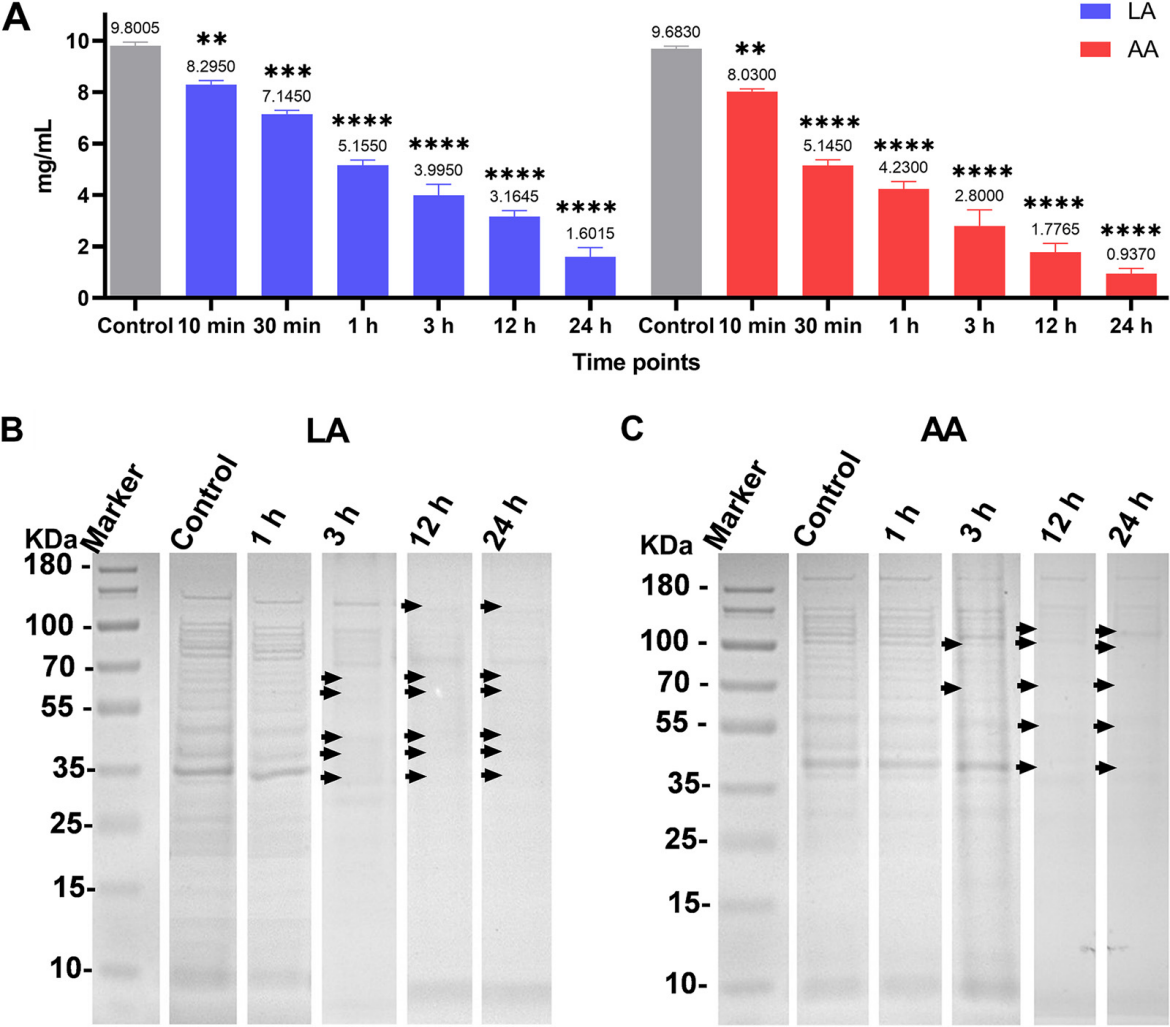
Effects of LA and AA on protein. (A) Protein concentrations after treatment with LA and AA. (B and C) SDS-PAGE patterns show the effects of LA (B) and AA (C) on the protein profile of A. baumannii. Membrane fractions were analyzed by 10% SDS-PAGE stained with Coomassie blue. A total of 20 μL of protein was loaded in each lane. **, *P < *0.01; ***, *P < *0.001; ****, *P < *0.0001.

An interruption effect on the proteins was observed for both LA and AA. After treating the protein samples extracted from A. baumannii with LA, the concentration decreased remarkably with prolonged treatment. A similar trend of reduction was obtained after treatment with AA (Fig. 8A).

The protein patterns of A. baumannii were also changed after exposure to LA and AA. As shown from the SDS-PAGE gels, the number of visible bands decreased remarkably from 3 h. The noticeable differences were missing bands between 70 and 35 kDa for LA and between 100 and 45 kDa for AA at 3 h. Impressively, nearly all bands were diminished at 12 h and 24 h (Fig. 8B and C).

### Effects of LA and AA on biofilm-related genes and DNA repair genes.

**FIG 9:**
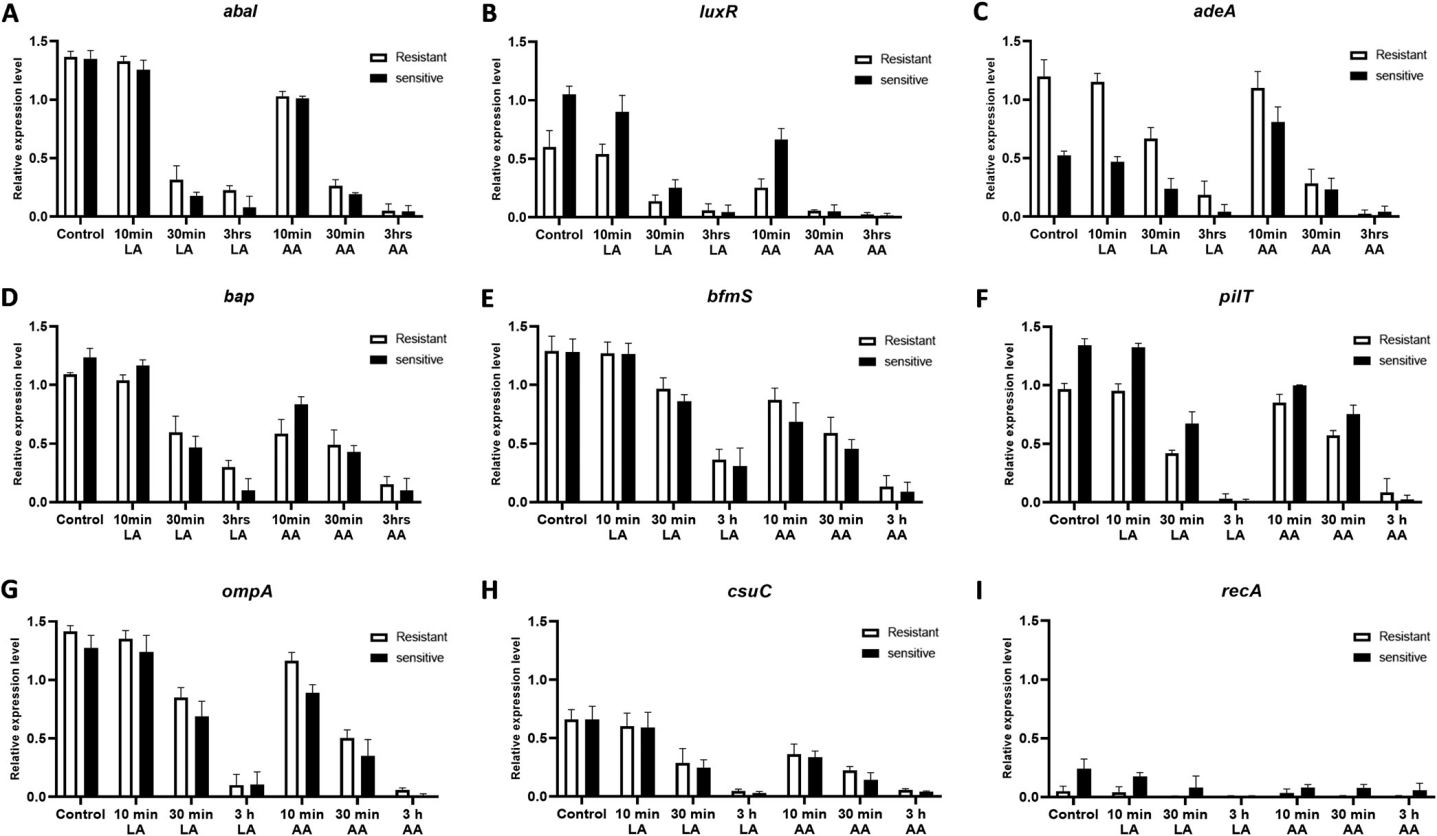
Effects of LA and AA on the expression of representative biofilm formation-related genes and DNA repair genes in A. baumannii. The expression levels of *abaI* (A), *luxR* (B), *adeA* (C), *bap* (D), *bfmS* (E), *pilT* (F), *ompA* (G), *csuC* (H), and *recA* (I) were examined after exposure to LA or AA for 10 min, 30 min, and 3 h. All of the experiments were repeated three times, and the results are shown as means ± SD.

In order to evaluate the effects of LA and AA on biofilm-related and DNA repair genes in A. baumannii, the expression levels of each gene among sensitive and resistant isolates were analyzed with real-time PCR. The selected representative genes were *abaI*, *luxR*, *pilT*, *bap*, *adeA*, *bfmS*, *csuC*, *ompA*, and *recA*. Based on the results, there were variations in gene expression among treated isolates which were in accordance with the differential patterns of the modes of action of LA and AA on biofilm formation. The longer the treatment with LA and AA, the greater the downregulation of all genes observed. The above results suggested that the whole genome of A. baumannii might be affected by LA and AA (Fig. 9).

### DNA interactions with LA and AA.

**FIG 10:**
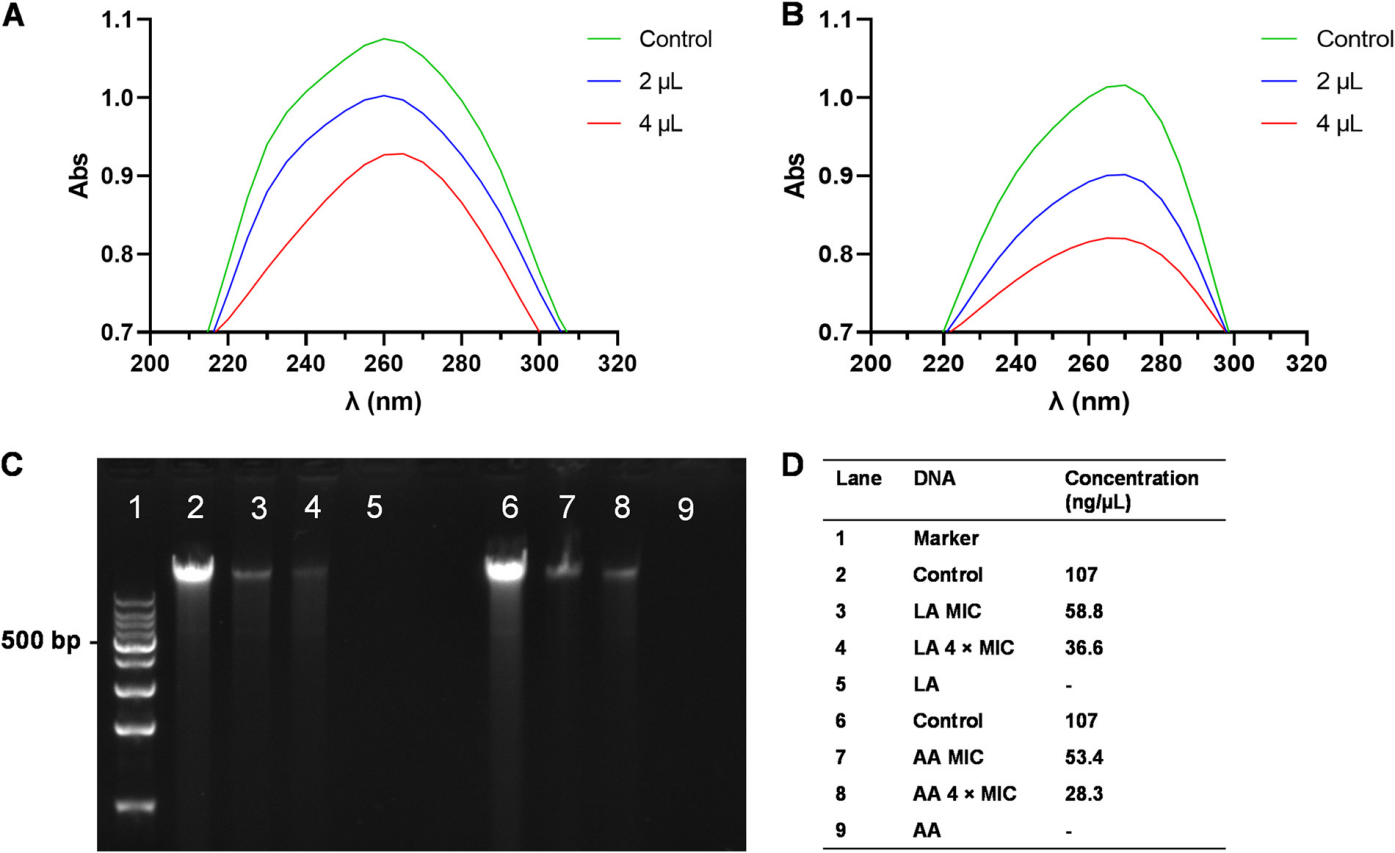
(A and B) DNA interaction with LA (A) and AA (B). (C and D) Agarose gel electrophoresis and final concentration analysis of A. baumannii DNA after direct treatment with different concentrations of LA and AA.

The interaction of bacterial DNA with LA and AA was detected to determine the mechanism of these bactericidal materials working on DNA. UV absorption spectroscopy is often used to investigate interactions between active compounds and DNA. The spectrum showed that LA and AA had maximum absorption peaks at 260 nm and 265 nm, respectively. However, the values of the peaks decreased dramatically after adding 2 μL and 4 μL of DNA to the reagents. These results indicated that the interaction with LA and AA caused damage to the bacterial DNA (Fig. 10).

Moreover, due to the damage of cell membranes and proteins, the decreasing gene expression trend might have the result of DNA and RNA leakage. Thus, the direct effect of LA and AA on DNA was examined. The quantity of DNA decreased with increasing LA and AA concentrations. After being treated by LA at the MIC and 4× the MIC, the concentration of DNA decreased from 107 ng/μL to 58.8 ng/μL and 36.6 ng/μL, respectively. Analogously, these values dropped to 53.4 ng/μL and 28.3 ng/μL after treatment with AA.

## DISCUSSION

A. baumannii can form biofilm on a wide range of abiotic and biotic surfaces (18), which is a significant virulence factor that contributes to colonization and eventually leads to invasive illnesses (5). Microbial cells within biofilms have shown 10 to 1,000 times more antibiotic resistance than planktonic cells (19), so biofilms act as reservoirs for disease recurrence (20). As a result, normal antibiotic administration does not prevent bacterial colonization, and high antibiotic doses are needed to suppress microorganisms that form biofilms.

Many treatment techniques have attracted interest from clinicians and scientists (21). Bacteriophages, antimicrobial peptides, monoclonal antibodies, and the clustered regularly interspaced palindromic repeats/CRISPR-associated protein system (CAS) are options for treating A. baumannii infections (22, 23). However, these techniques have limitations, including cytotoxicity, moderate activity, enzymatic degradation, high cost, and a low volume of productivity (21). Therefore, natural alternatives, such as probiotics and their derivatives, are better choices to control biofilm formation on medical equipment and related infections.

As reported earlier, *Bifidobacterium* and *Lactobacillus* are the most common microbial genera used as probiotics (24). However, lactobacilli have a greater abundance, with more than 200 identified species enrolled in the List of Prokaryotic names with Standing in Nomenclature (LPSN) (25), as well as higher phylogenetic and phenotypic levels and a greater diversity of bacteriocins compared with *Bifidobacterium* species. Furthermore, L. rhamnosus has been reported to be able to inhibit the adhesion of Escherichia coli, Klebsiella pneumoniae, Pseudomonas aeruginosa, and Staphylococcus aureus during the process of biofilm formation (14, 26, 27). Indeed, among the tested lactobacilli in our study, L. rhamnosus showed the best performance against A. baumannii biofilm, not only in terms of its antiadherent effect, but also its antimaturation effect.

The BSs produced by L. rhamnosus could be used as active compounds to inhibit the pathogen biofilms (15). LA, AA, galactose, l-rhamnose, and rhamnolipid are the currently known BSs with activity against bacteria and fungi (28–33). In this study, LA and AA were the most effective agents in obstructing the formation of A. baumannii biofilm. Since bacterial adhesion is the critical step in the progression of biofilm formation, the antiadherent effect of BSs reduces or prevents bacterial adhesion and growth, which represents the antimicrobial nature of these probiotics (34). As expected, a strong bactericidal effect of LA and AA was detected in our work, resulting in a significant reduction in biofilm formation. Surprisingly, A. baumannii cells were disrupted by both LA and AA from very early time points, which confirmed that these two less harmful compounds were growth inhibitors and possessed efficient antimicrobial properties (35, 36). In addition, the remarkably decreased motility of A. baumannii, which was a reflection of bacterial activity and an attribute of pathogen colonization, also supports the presence of an antibacterial effect (37, 38).

Weak acids have been reported to be an alternative approach to control bacterial growth, since they have the ability to penetrate the full depth of microcolonies within 2 to 3 h and kill the bacteria (39, 40). As observed by CLSM, A. baumannii cells were interrupted by LA and AA in an even shorter time. The main roles of the cell membrane are to protect the inner components of the cell and to act as a selectively permeable barrier, as well as producing energy. Thus, the penetration of antimicrobial agents and their direct actions on the cell membrane are the initial step in the destruction of bacterial cells (41). As monitored by TEM and ATPase activity, the disturbance of the integrity of the cell envelope by LA and AA leads to severe damage in A. baumannii. The decreased cellular ATPase activity of Na^+^/K^+^-ATPase and Ca^2+^/Mg^2+^-ATPase can reduce energy metabolism (42, 43), thereafter aggravating the damage to A. baumannii cells. This also leads to an imbalance in osmotic pressure, which triggers the leakage of the cell content, apoptosis, and the disruption of cellular metabolism (44, 45). Therefore, changes in energy metabolism may be one of the reasons for the antimicrobial efficacy of LA and AA. Moreover, the breaking down of the membrane is a cell death pathway caused by cell wall-targeting antimicrobials (46).

Additionally, the internalized weak acids dissociate, acidifying the cytoplasm, which in turn can cause acid-induced protein unfolding and DNA damage (40, 47). Indeed, LA and AA disrupted the cytoplasm and could cause protein lysis, which was attributed to the effect of acids as protein-denaturation agents (48).

The expression of biofilm-related genes was also affected by LA and AA. The frequently known biofilm formation factors include motility, the chaperone usher (CU) system, efflux pump (EP), quorum sensing (QS), mature biofilm maintenance, and outer membrane proteins. Pili biosynthesis is mediated by the expression of the CU system, which is required for twitching motility (49). The maturation, maintenance, and development of biofilm are all affected by *bap*, which is regulated by the two-component system BfmRS (50). Furthermore, the *omp* gene plays a significant role in cell membrane integrity and increases cell adhesion (51). Another virulence factor is the EP system, which has antibiotic and antibacterial resistance functions (52, 53), and also regulates the virulence factors associated with the expression of QS function (54, 55). Furthermore, RecA is the most important enzyme in homologous recombination and DNA repair in the fight against stressors such as DNA-damaging compounds, antibiotics, and acids (56). As shown by our results, the expression of all of the above-mentioned genes was decreased by LA and AA. Impressively, these two BSs could interact with DNA, and the downregulation of the genes was contributed to the decrease in DNA.

Since LA and AA can not only break A. baumannii cells but also lyse the outer membrane, proteins, and DNA, which are the components of the biofilm matrix, it was not surprising that the already-formed biofilm was erased completely.

In conclusion, this study mainly aimed to use probiotics as alternatives to eradicate and prevent biofilm formation. LA and AA had a significant inhibitory effect on A. baumannii biofilm and cells. These effects were achieved by several mechanisms, including the disruption of the cell envelope membrane, protein lysis, the reduced expression of biofilm-related genes, and the destruction of bacterial DNA. Thus, probiotics can be utilized as novel biocides to block and decrease biofilm formation and microbial contamination in medical equipment and during the treatment of infections.

## MATERIALS AND METHODS

### Bacterial strains and culture conditions.

The experiment included 70 isolates of A. baumannii collected from the First Affiliated Hospital of Xi’an Jiaotong University and Shaanxi Provincial People’s Hospital. All isolates were cultured in Luria-Bertani (LB) medium (Beijing Land Bridge Technology Co., Ltd., Beijing, China) at 37°C and stored at −80°C in 20% glycerol.

### Biofilm antimaturation assay of probiotics.

The inhibitory effect of *Lactobacillus* spp. against the biofilm of A. baumannii was observed by microtiter well plate biofilm assay. Strains of A. baumannii and *Lactobacillus* spp. were cultured in LB medium and grown to the stationary phase with shaking at 200 rpm. The bacterial suspensions of A. baumannii and *Lactobacillus* spp. were added to each well with final concentrations of 5 × 10^5^ CFU/mL and 1 × 10^8^ CFU/mL, respectively. A total of 200 μL of phosphate-buffered saline (PBS) was added to the first well as a negative control. Additionally, 200 μL of A. baumannii suspension was set as the positive control in the second well. After overnight incubation at 37°C, the planktonic bacteria were removed and the wells were washed with PBS. Then, 200 μL of 1% crystal violet was added to each well and the contents were kept at room temperature for 10 min, followed by washing with PBS. After drying, 200 μL of 95% ethanol was added to each stained well, and the plate was incubated for 15 min at room temperature to solubilize the dye. The optical density of each well was recorded at 570 nm. All of the tests were carried out in triplicate, and the results are given as the means of three replicates.

### Biofilm antiadherence assay of probiotics.

The antiadherent effect was tested in the same way as the antimaturation assay, but 100 μL of *Lactobacillus* spp. was added to each well and incubated for 24 h in advance. After washing with PBS, 100 μL of A. baumannii was added and the mixture was incubated at 37°C for another 24 h. The wells were washed with PBS and stained with crystal violet, followed by the measurement of the OD_570_.

### Antibiofilm effects of the major components of the biosurfactants derived from L. rhamnosus.

Five major L. rhamnosus components were chosen to test the antibiofilm and antibacterial effects against A. baumannii. These compounds were LA, AA, l-rhamnose monohydrate, d-(+)-galactose, and rhamnolipids.

The 2-fold dilution method in Mueller-Hinton broth (MHB; Oxoid, United Kingdom) was used to test the antimaturation and antiadherent effects of d-(+)-galactose, LA, rhamnolipids, and l-rhamnose monohydrate at concentrations of 500, 250, 125, 62.5, 31.2, 15.6, 7.8, 3.9, 1.95, 0.97, 0.48, and 0.24 mg/mL, respectively, and of AA at concentrations of 100, 50, 25, 12.5, 6.25, 3.125, 1.56, 0.78, 0.39, 0.195, 0.097, and 0.048%. To evaluate the antimaturation activity, 100 μL of A. baumannii at the final concentration of 5 × 10^5^ CFU/mL was cocultured with 100 μL of different BSs and statically incubated at 37°C for 24 h. To evaluate the antiadherent activity, 200 μL of each BS was seeded into 96 wells and incubated at 4°C for 24 h. Later, the BSs were removed and 200 μL of A. baumannii suspension was added and incubated at 37°C for another 24 h.

### Determination of MICs for LA and AA.

MICs of LA and AA were assessed to identify the most appropriate concentrations for antibiofilm effects. Susceptibility to LA and AA was assessed using the broth microdilution method according to the Clinical and Laboratory Standards Institute guidelines (57) with some modifications. A. baumannii was cultured in MHB and tested at the concentration of 5 × 10^5^ CFU/mL. LA and AA were determined at a range of final concentrations of 8 to 0.5 mg/mL and 0.8% to 0.05%, respectively. The results were recorded after incubation at 37°C for 24 h. Experiments were carried out with three replicates.

### Biofilm clearance effects of LA and AA at different time points.

The clearance abilities of LA and AA against preformed biofilms were assessed. Overnight A. baumannii strains were diluted in LB to an OD_600_ of 0.2. Then, 200 μL of A. baumannii suspension was seeded into 96 wells and statically incubated at 37°C for 24 h. After incubation, the wells were washed with PBS to remove any unbound cells, and 100 μL of LA and AA at concentrations of 8 to 0.5 mg/mL and 0.8% to 0.05%, respectively, was added. The plates were then incubated at 37°C for 6, 12, and 24 h.

### Bactericidal curve of A. baumannii by LA and AA.

An overnight culture of A. baumannii was adjusted to an OD_600_ of 0.2, followed by a 1:100 dilution, which was further cultured to obtain the bacterial number of 10^7^ CFU/mL. The bacterial suspensions were treated with different concentrations of LA and AA at the MIC, 2× the MIC, and 4× the MIC and incubated at 37°C with shaking at 200 rpm. A total of 100 μL of the treated bacterial samples was taken from the time points of 0, 3, 6, 9, 12, 18, and 24 h and then plated on agar plates for counting the colonies (58).

### Investigation of the bactericidal effects of LA and AA by CLSM.

An overnight suspension of A. baumannii was 1:100 diluted in 5 mL of LB broth for 3 h, and the culture was adjusted to an OD_600_ of 0.2. Later, bacterial suspensions were centrifuged at 4°C, 2,600 × *g*, for 10 min. The pellets were resuspended in 100 μL of PBS containing the MIC of LA or AA and incubated for 10 min, 30 min, 1 h, 3 h, 6 h, and 12 h at 37°C. Thereafter, bacterial suspensions were stained with SYTO9 and propidium iodide for 20 min at room temperature, followed by centrifugation at 4°C, 2,600 × *g*, for 10 min. The pellets were washed with PBS at 4°C two times and then thoroughly resuspended in PBS containing 70% glycerol. Finally, the bactericidal effects of LA and AA were recorded using a confocal laser scanning microscope (TCS SP8 STED 3×, Leica Microsystems, Nanterre, France). Images were captured at 63× magnification. A group with untreated samples was used as the control.

### Inhibition of twitching motility by LA and AA.

The twitching motility of the treated samples was tested using MHB medium containing 0.8% (wt/vol) agar. The medium was supplemented with 1/2× the MIC of LA or AA and was poured into the plates. After solidification, the fresh bacterial cultures were inoculated into the bottom of the plates using a toothpick, as mentioned in prior work (59). The plates were then incubated at 37°C for 48 h. After incubation, the agar was removed and the plates were stained using 1% crystal violet before being imaged with a PowerLook 2100 XL-USB scanner (UMAX, USA). The tests were repeated three times. Each isolate's twitching motility was categorized as follows: not motile (<5 mm), intermediate (5 to 20 mm), or strong (>20 mm).

### TEM.

TEM samples were prepared as previously described (60), with modifications. After treatment with LA or AA in PBS for 3 h, the samples were centrifuged at 2,600 × *g* at 4°C for 5 min. The pellets were fixed in 2.5% glutaraldehyde (SCRC, China) at 4°C for 2 h, followed by washing with PBS for 10 min, and then fixed in 1% osmium tetraoxide (Johnson Matthey, England) at 4°C for 2 h. The samples were dehydrated with ethanol and embedded in Epon 812 epoxy resin (SPI-Chem, USA) at 60°C for 24 h. Thin-section samples of 50 to 70 nm were made using an LKB-Vultratome apparatus (LKB, Sweden). After staining with uranyl acetate and lead citrate for 15 min, the samples were finally observed and photographed under the transmission electron microscope (Hitachi H-7650, Japan).

### Na^+^/K^+^-ATPase and Ca^2+^/Mg^2+^-ATPase activity tests.

Overnight cultures of A. baumannii isolates were adjusted to an OD_600_ of 0.2 and incubated with the MIC of LA or AA for 30 min, 1 h, 3 h, 6 h, and 12 h. Untreated samples were used as the control. After incubation, the Na^+^/K^+^ ATPase and Ca^2+^/Mg^2+^-ATPase activities were determined following the manufacturer’s instructions for the Ultra Trace sodium potassium ATPase test kit (Nanjing Jiancheng Bioengineering Institute, Nanjing, China). Approximately 100 μL of each sample was combined with reagents B and E and incubated at 37°C for 30 min. The mixture was centrifuged at 4°C, 2,600 × *g* for 10 min. The supernatant was then extracted for the measurement of the phosphorus concentration. After adding the phosphorus fixation agent and maintaining it at room temperature for 5 min, the absorbance of each tube was measured at 660 nm.

### Total protein extraction.

Overnight bacterial cultures were incubated in LB medium at 37°C with agitation. Cells were harvested by centrifugation at 4,700 × *g* (10 min at 4°C) and then washed with PBS. Bacterial cells were disrupted by sonication (three bursts of 30 s each) using a Vibra-cell VCX 750 probe sonicator with a CV 26 probe (tip diameter of 3 mm; Sonics & Materials, Newtown, CT, USA) at a frequency of 20 kHz. The sample vial was kept in an ice-water bath to prevent significant heating in the sample during sonication. Cell debris was removed by centrifugation at 4°C, 10,000 × *g*, for 10 min.

Protein concentrations of treated samples were determined using a bicinchoninic acid kit (Dingguo Changsheng Biotechnology Co., Ltd., Beijing, China) according to the manufacturer's instructions. Briefly, samples were incubated up to 2 h at 37°C, with 150 μL of sample plus 150 μL of the working reagent. The absorbance was measured at 562 nm using a Multiskan GO microplate reader (Thermo Scientific, Vantaa, Finland).

### Polyacrylamide gel electrophoresis.

After adding 5 × protein sample loading buffer (Epizyme, Shanghai, China), the samples were boiled for 10 min at 95°C. Twenty-microliter aliquots of protein samples treated by LA or AA at the respective MIC were loaded into 10% SDS-PAGE gels (Epizyme). A Thermo Scientific PageRuler Prestained Protein Ladder was used as molecular weight marker ranging from 10 −180 kDa. Electrophoresis was performed at 80 V for 30 min and then at 100 V for 60 min. After electrophoresis, gels were stained with Coomassie blue stain for 2 h.

### RNA preparation and real-time PCR.

The freshly formed biofilm was treated with LA and AA at 1/2 MIC and incubated at 37°C for 10 min, 30 min, and 3 h. Subsequently, the RNA of the biofilm was extracted using the RNAprotect Bacteria Reagent (Qiagen, Hilden, Germany) and RNAprep Pure kit (Tiangen Biotech Co., Ltd., Beijing, China) according to the manufacturer’s protocols.

The procedure for detecting gene expression was performed as reported previously, with some modifications (61). The cDNAs were reverse-transcribed from DNA-free total RNAs by using a RevertAid First Strand cDNA synthesis kit (Thermo Scientific) with a random hexamer primer. AB5116 was set as the reference strain and was used as a control to evaluate the gene expression levels of resistant and sensitive strains in the clinic. The 16S rRNA, which is a housekeeping gene, was used for standardization. The real-time PCR was performed using TB Green Premix Ex Taq (Til RNaseH Plus; TaKaRa Bio, Inc., Beijing, China) in a final volume of 20 μL. Additionally, quantitative PCR (qPCR) was conducted using an Agilent Mx3005P qPCR system (Agilent Technologies, Santa Clara, CA, USA) under the conditions of initial incubation at 95°C for 2 min, followed by 35 cycles of 95°C for 30 s, 55°C for 30 s, and 72°C for 1 min. The 2^−ΔΔ^*^CT^* threshold cycle method was used to examine the real-time PCR results. The primers used in this study are listed in Table 1. All assays were carried out in triplicate.

**TABLE 1:** qPCR primers for biofilm-related genes

Genes	Primer sequences (5′–3′)	Product size (bp)	Reference
*ompA*	F: TACCCTAACGCTACTGCACG R: TCGGTTGATCCCAAGCGAAA	172	This study
*abaI*	F: CCGCTACAGGGTATTTGTTGAA R: GGTTGTGTGGTGGGTAGT	159	This study
*bfmS*	F: ACCAACGCGCACAAGAATAC R: TTTGCTGCTTCCCAGGTT	93	This study
*luxR*	F: TATTGGGACCCGAGGAATGC R: GCTGGAATGCACTGTTTGAG	115	This study
*csuC*	F: CTTGTTACACCTGTGATGGC R: GCATCGGTCTTACCCGTAT	116	This study
*adeA*	F: CAGTTCGAGCGCCTATTTCT R: TATTGGTATCGCCCTGACCG	81	This study
*pilT*	F: GCGAGCGACTAAGCCTTTTA R: GATTGGTATTCCGGCTATTCGT	130	This study
*bap*	F: AGGGAACTTCTGCAAAACTTTC R: CAGACGTATGACTGCATTGGT	108	This study
*recA*	F: CGGTTATGCGTCTTGGTG R: AGACATCGCATTGGGGATTG	87	This study
16s rRNA	F: CAGCTCGTGTCGTGAGATGT R: CGTAAGGGCCATGATGACTT	150	62

### DNA interaction with LA and AA.

The genomic DNA of A. baumannii was extracted from an overnight culture using the TIANamp bacterial DNA kit (Tiangen Biotech Co., Ltd.). A total of 2 μL and 4 μL of bacterial DNA was incubated with the respective MIC of LA or AA in a total volume of 1.5 mL at 37°C for 20 min. The UV absorption was measured at 210 to 310 nm using a UNICO UV-2100 spectrophotometer (UNICO, Shanghai, China).

### Direct treatment of DNA.

The direct effects of LA and AA on DNA were investigated. A total of 20 μL of the genomic DNA was treated with LA and AA at the MICs and 4× the MICs. Finally, the changes in DNA concentrations were observed with a microplate reader (Thermo Scientific), and the samples were analyzed by gel electrophoresis.

### Statistical analysis.

All statistical analyses were performed using GraphPad Prism (version 8.0; San Diego, CA, USA). The effects of probiotics and the derivative BSs LA and AA against A. baumannii biofilms were analyzed using Dunnett’s one-way analysis of variance (ANOVA). The effects on the bactericidal and protein lysis of LA and AA were determined by Dunnett’s one-way ANOVA. The activities of Na^+^/K^+^-ATPase and Ca^2+^/Mg^2+^-ATPase were analyzed by paired *t* tests. A *P* value of <0.05 was considered statistically significant for all tests.
